# A novel bivariate nomogram for predicting sequela vaginal lesions after surgery in patients with HPV-associated cervical cancer

**DOI:** 10.3389/fonc.2025.1587520

**Published:** 2025-09-26

**Authors:** Yueheng Liu, Yue Shi, Yunqiang Zhang, Keqin Hua, Jingxin Ding

**Affiliations:** Department of Gynecology Oncology, The Obstetrics and Gynecology Hospital of Fudan University, Shanghai, China

**Keywords:** vaginal intraepithelial neoplasia, vaginal carcinoma, persistent hpv infection, cervical cancer, predictive models

## Abstract

**Objective:**

To study and predict the risk of further vaginal lesions after surgery in patients with HPV-associated cervical cancer.

**Methods:**

Medical records of women who underwent surgery for cervical cancer between January 2018 and December 2022 at the Obstetrics and Gynecology Hospital of Fudan University were analyzed. Incidence and genotype of persistent HPV infection were recorded and patients with further vaginal lesions were analyzed for clinicopathological risk factors. Of the patients, 70% were randomly grouped into a training cohort, and predictive prognostic models for vaginal lesions were constructed through machine learning. The model with the highest area under the receiver operator curve (AUC) was screened out in the testing cohort. The nomogram and its calibration curve presented the risk of sequela vaginal lesions. R 4.2.0 software was used for all data processing.

**Results:**

Within five years after surgery, 29.94% of patients remained persistently infected with HPV, with annual rates fluctuating around 22%. In addition, 10.2% of patients were diagnosed with vaginal intraepithelial neoplasia (VaIN), and 320 cases (78.35%) were low-grade squamous intraepithelial lesions (LSIL). The annual incidence of vaginal lesions decreased gradually from 6.97% in the first year (Y1) to 2.96% at year 5 (Y5). Ovarian preservation (OP) during hysterectomy and adenocarcinoma histology were found to be protective from further vaginal lesions, while elder age, FIGO stage II, and positive vaginal incision margin were significant risk factors. As for persistent HPV infection, both single and multiple genotype remarkably increased the risk of vaginal lesions, and a α-9 HPV infection (OR = 18.20) brought higher risk than non-α-9 HPV (OR = 11.76). Then we built three predictive models; multiple logistic regression was optional, with its AUC at 0.7955 in the ROC curve.

**Conclusion:**

The predictive model constructed in our study could identify populations at high risk of vaginal lesions and precisely guide clinical interventions.

## 1 Introduction

Cervical cancer is the second most common gynecologic malignancy worldwide and the most commonly diagnosed gynecological malignancy in developing countries like China (1). Relevant research showed there were 150700 women diagnosed with cervical cancer and 55700 who died from it in 2022 in China (2). Patients with cervical cancer were mainly treated with radical hysterectomy plus pelvic lymph node dissection if at an early stage. Due to the use of cytology and HPV co-testing becoming widespread among the sexually active female population in China, over half of cervical cancer cases are diagnosed at Stage I, with a five-year survival rate exceeding 80% (3). However, local recurrence for these patients is not satisfactory, with a five-year recurrence rate of 10% to 20%. This could present as newly diagnosed vaginal cancer or vaginal intraepithelial neoplasia (VaIN) due to the persistence of high-risk human papillomavirus (HR HPV) and the subsequent malignant transformation of vaginal epithelial cells (4).

Previous studies reported that vaginal lesions could be found in over 10% of women hysterectomized due to cervical carcinoma. The HPV genotype test and Cytology co-test are still needed to routinely screen for vaginal disease (5, 6). In the past decade, the volume of colposcopic examinations rose rapidly, and the diagnosis of VaIN has increased steadily. In our center, the annual volume of colposcopic examination grew from 30399 cases in 2015 to 53800 in 2024. In 2025, 4501 cases of VaIN were diagnosed at our center. As for VaIN patients without a history of cervical cancer, low-grade squamous intraepithelial lesions (LSIL) could still be subjected to follow-up, while high-grade vaginal squamous intraepithelial lesions (HSIL) could need surgical excision or carbon dioxide laser treatment. For patients who undergo hysterectomy for cervical intraepithelial neoplasia (CIN) grade 3 or cervical cancer, as the epithelial tissue buried in the vaginal vault scar cannot be effectively evaluated, thorough surgical biopsy is preferred under this circumstance, which can cause patients tremendous pressure both physically and mentally (7).

It seemed critical to screen for risk factors of further vaginal disease to help predict and precisely diagnose sequela vaginal disease after hysterectomy. This study took place at the Obstetrics and Gynecology Hospital of Fudan University, the largest obstetrics and gynecology medical center in China. We aimed to explore the risk factors associated with sequela vaginal disease after hysterectomy and calculated risk scores to predict and tailor our clinical surveillances and further interventions of hysterectomized women due to cervical carcinoma.

## 2 Materials and methods

### 2.1 Patient accrual

**Figure 1:**
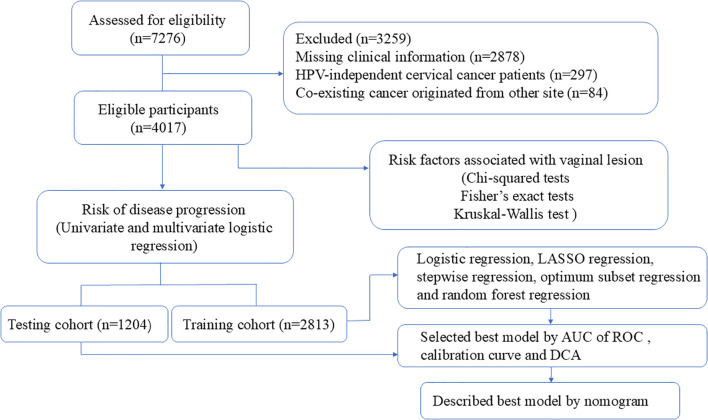
Study design and workflow.

After obtaining institutional review board approval from the Obstetrics and Gynecology Hospital of Fudan University (Ob&Gyn Hospital) (No.2024-81) on 24 April 2024, a computer-based search of the patients who underwent radical trachelectomy or hysterectomy between January 2018 to January 2022 at our hospital was carried out to retrieve cases. The inclusion criterion was patients who (1) underwent radical trachelectomy or hysterectomy at our hospital (2) with a postoperative pathology-confirmed invasive cervical carcinoma. The exclusion criteria included patients with (1) missing clinical information, (2) who were HPV negative before surgery, or (3) with co-existing cancer that originated from other sites. Finally, 4017 patients were enrolled, including those who desired to preserve their fertility and were surgically treated with radical trachelectomy (**Figure 1**).

### 2.2 Explanatory and outcome variables

Both clinical and pathological data were obtained from medical records. The explanatory clinicopathological variables included age at diagnosis, histopathological diagnosis (WHO 2014), tumor size, lymph node metastasis (LNM), depth of stromal invasion (DI), parametrial invasion, lower uterine corpus invasion (LUCI), incision margin, vaginal involvement, FIGO stage (2019), surgical modalities (including vagina elongation, fertility-sparing, ovarian preservation, and para-aortic lymph node dissection), and follow-up information (including radiotherapy, chemotherapy, and HPV infection status). The outcome variable was the condition of the vaginal epithelium, based on the results of colposcopic pathology or LCT. Although patients with abnormal LCT results are encouraged to seek further examination, a small number of patients refuse as a personal choice, and the LCT result was recorded as their outcome under the circumstance. The endpoint of follow-up was December 2022, and the dates of LCT and colposcopic surgery were obtained from our medical record system.

Chemotherapy regimen included sequential chemotherapy and concurrent chemotherapy during adjuvant radiotherapy. HPV genotyping was performed using the Bioperfectus Technologies human papillomavirus genotyping real-time polymerase chain reaction (PCR) kit (Bioperfectus Ltd, Jiangsu, China), which included 21 different types of HPV, with eighteen high-risk HPV types (16, 18, 26, 31, 33, 35, 39, 45, 51, 52, 53, 56, 58, 59, 66, 68, 73, and 82) and three low-risk HPV types (6, 11, and 81). Persistent HPV infection meant patients who were still infected with HPV one year post-operation.

### 2.3 Descriptive statistics and variable selection

Continuous variables (age and tumor size) were found to be abnormally distributed, thus a Kruskal-Wallis test was used to analyze these variables. A chi-squared test and Fisher’s exact test were applied for categorical variables. Baseline results that (shown in **Table 1**) were generated using R package “tableone”. Univariate and multivariate logistic regression estimated the odds ratio (OR) and 95% confidence interval (CI) to analyze the risk factors for postoperative vaginal lesions. The collinearity between multiple genotype HPV infection and α-9 HPV (including HPV 16, 31, 33, 35, 52, 58, 67) infection, multiple HPV infection and vaginal lesion, and α-9 HPV infection and vaginal lesion were calculated by R package “autoReg”.

**Table 1:** Clinicopathological characteristics of the cohort and potential risk factors for sequela vaginal lesions.

Clinical characteristics			N=4017	Vaginal lesion		P
				No	Yes	
Age (year)(median)			49	48	50	0.0028***
Fertility-sparing surgery (%)	No		3937(98.01)	3539 (89.89)	398 (10.11)	
	Yes		80(1.99)	67 (83.75)	13 (16.25)	0.1079
Ovarian preservation (%)	No		2649(65.94)	2358 (89.01)	291 (10.99)	
	Yes		1368(34.06)	1248 (91.23)	120 (8.77)	0.0325**
Vaginal elongation (%)	No		3716(92.50)	3342 (89.94)	374 (10.06)	
	Yes		301(7.50)	264 (87.71)	37 (12.29)	0.2594
Para-aortic lymph node dissection (%)	No		3604(89.72)	3235 (89.76)	369 (10.24)	
	Yes		413(10.28)	371 (89.83)	42 (10.17)	1
FIGO stage (%)	I		2681(66.74)	2415 (90.08)	266 (9.92)	
	II		768(19.12)	671 (87.37)	97 (12.63)	
	III		568(14.14)	520 (91.55)	48 (8.45)	0.0293**
Histology (%)	Squamous carcinoma		3108(77.37)	2777 (89.35)	331 (10.65)	
	Adenocarcinoma		549(13.67)	511 (93.08)	38 (6.92)	
	Adenosquamous carcinoma		284(7.07)	249 (87.68)	35 (12.32)	
	Others		76(1.89)	69 (90.79)	7 (9.21)	0.0354**
Tumor size (cm)(median)			1.6	1.6	1.5	0.2815
DI (%)	Superficial 1/3		2075(51,65)	1856(89.45)	219(10.55)	
	Middle 1/3		508(12.65)	461 (90.75)	47 (9.25)	
	Deep 1/3		1434(35.70)	1289(89.89)	145 (10.11)	0.6766
LUSI (%)	Negative		3527(87.80)	3161(89.62)	366(10.38)	
	Positive		490(12.20)	445 (90.82)	45 (9.18)	0.461
Parametrial invasion (%)	Negative		3731(92.88)	3349(89.76)	382(10.24)	
	Positive		286(7.12)	257 (89.86)	29 (10.14)	1
Vaginal incision margin status (%)	Negative		3758(93.55)	3389(90.18)	369(9.82)	
	LSIL		31(0.77)	25 (80.65)	6 (19.35)	
	HSIL		97(2.42)	74 (76.29)	23 (23.71)	
	Cancer		131(3.26)	118 (90.08)	13 (9.92)	<0.0001***
Vaginal invasion (%)	Negative		3202(79.71)	2888(90.19)	314(9.81)	
	Positive		815(20.29)	718(88.10)	97(11.90)	0.0896*
LNM (%)	Negative		3441(85.66)	3081 (89.54)	360 (10.46)	
	Positive		576(14.34)	525 (91.15)	51 (8.85)	0.2695
HPV infection (%)	Negative		2814(70.05)	2740 (97.37)	74 (2.63)	
	Single HPV infection	Non α-9 HPV infection	577(14.36)	444(76.95)	133(23.05)	
		α-9 HPV infection	281(7.00)	209(74.38)	72(25.62)	<0.0001***#
	Multiple HPV infection	Without α-9 HPV	95(2.37)	66(69.47)	29(30.53)	
		With α-9 HPV	250(6.22)	147(58.8)	103(41.2)	<0.0001***#
Chemotherapy (%)	Unreceived	2482(61.79)	2224(89.61)	258(10.39)	
	Received	1535(38.21)	1382(90.03)	153 (9.97)	0.7034
Radiotherapy (%)	Unreceived	2294(57.11)	2064(89.97)	230(10.03)	
	Received	1723(42.89)	1542(89.50)	181 (10.50)	0.6578

*P<0.1, **P<0.05, ***P<0.01.

#Compared with patients with negative HPV.

### 2.4 Model construction, selection, and calibration

The cohort of 4017 patients were randomly divided into training and testing clusters (2813 and 1204 patients, respectively) using “caret” R package. To identify potentially significant factors affecting the risk of vaginal lesions, we performed univariate logistic regression analyses on the training cohort. Factors with p-values < 0.1 were considered candidates for inclusion in the multivariate models. Then, we employed multiple methods to construct risk scores in the training cohort, including Least Absolute Shrinkage and Selection Operator (LASSO) regression, stepwise regression, optimum subset regression, random forest regression, and logistic regression. Each method was chosen for its unique strengths in handling high-dimensional data, avoiding overfitting, and capturing complex interactions. The method that yielded the best AUC of ROC, the degree of agreement with the 45°curve of the calibration curves and decision curve analysis (DCA) in the testing cohort, was selected as optional for vaginal lesion risk prediction. After that, a nomogram was established based on a new model to present the probability of vaginal lesions in future cases.

### 2.5 Software and tools

All data processing and analyses were conducted using R 4.3.3 software leveraging a range of specialized packages including “grid”, “forestploter”, “ggplot2”, “reshape”, “ggalluvial”, “dplyr”, “ggh4x”, “tidyr”, “ggrepel”, “patchwork”, “tableone”, “lattice”, “caret”, “compareGroups”, “glmnet”, “randomForest”, “ggthemes”, “leaps”, “autoReg”, “rcompanion”, “pROC”, “riskRegression”, “rms”, “riskRegression”, and “rmda”. These packages offered powerful and flexible tools for statistical modeling, visualization, and interpretation, enabling us to conduct our analyses with precision and efficiency.

## 3 Results

### 3.1 Patient characteristics

4017 patients were finally enrolled in our study, with their clinicopathological characteristics shown in **Table 1**. The median age was 49 years old (yo) with the peak distributed between 43 and 54 yo. 66.74% (2682/4017) of cases were diagnosed at stage I, with 77.37% of cases diagnosed as squamous carcinoma, followed by 13.67% with adenocarcinoma and 7.07% with adenosquamous carcinoma. Only 2% of the cohort (80/4017) accepted fertility-sparing surgery, 7.5% underwent vaginal elongation during surgery, and more than 1/3 of the cases at reproductive age preserved their ovaries.

6.45% (259/4017) of the patients had a positive incisional margin, among whom nearly half (131, 50.58%) had cancer, 37.45% (97 cases) had HSIL, and 11.97% (31 cases) had LSIL.

Nearly half (1724/4017,42.89%) of the patients underwent radiotherapy and 1536 (38.21%) accepted chemotherapy as an adjuvant therapy.

### 3.2 Persistent HPV infection and sequela vaginal lesions after surgery

**Figure 2:**
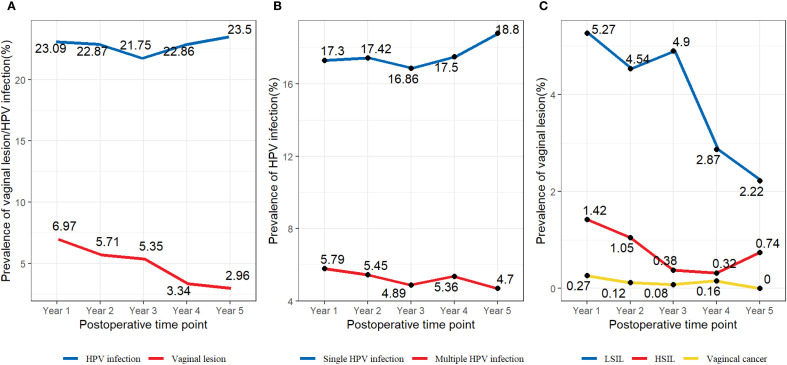
**(A)** Prevalence of specific HPV genotypes among the vaginal LSIL population. **(B)** Prevalence of specific HPV genotypes among the vaginal HSIL population. **(C)** Prevalence of specific HPV genotypes among the vaginal cancer population.

**Figure 3:**
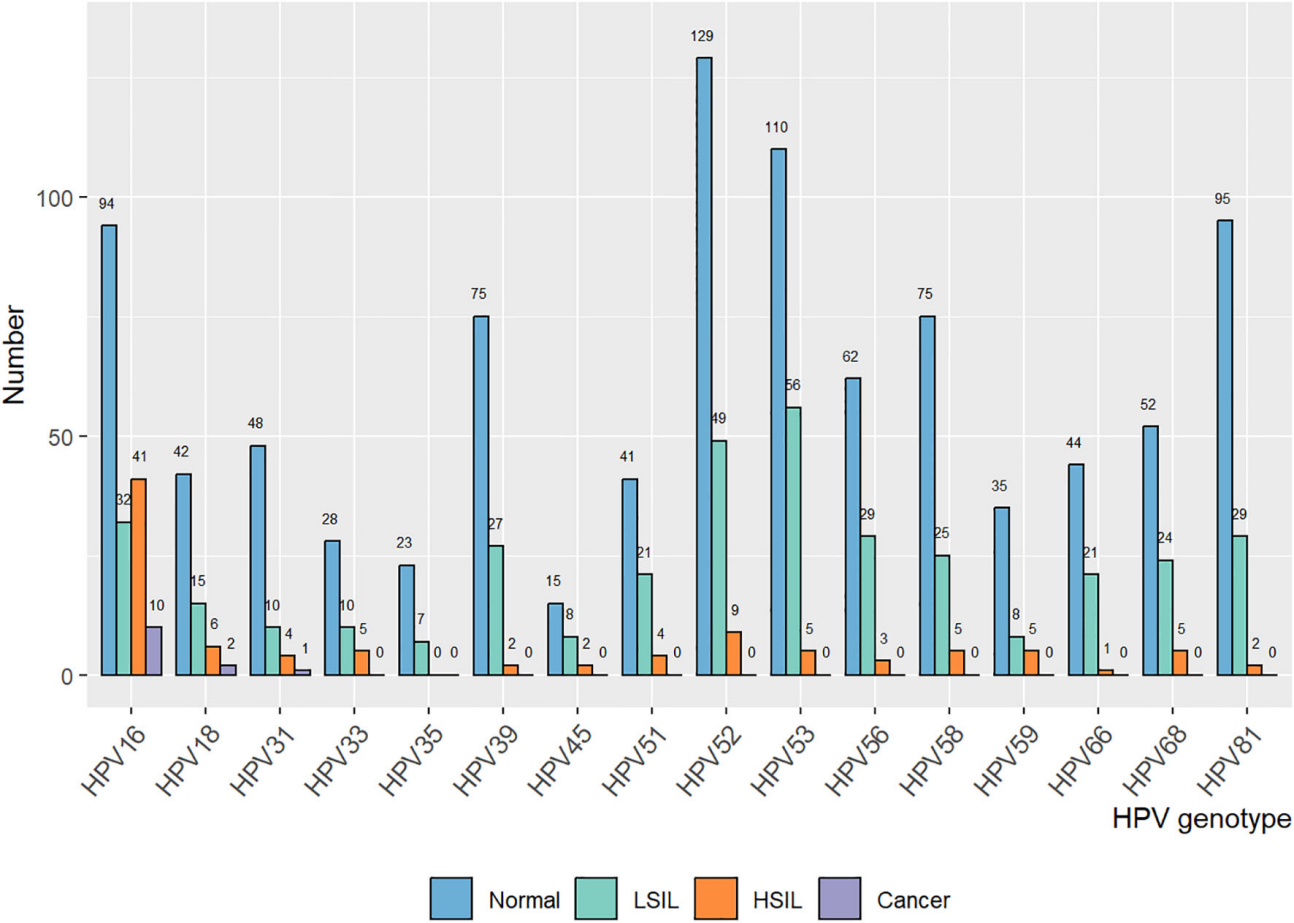
Association of HPV genotype and vaginal disease progression in the patient cohort with a single HPV infection.

We retrospectively focused on the five-year postoperative period, and persistent HPV infection was found in 1203 of 4017 patients (29.95%). The annual HPV infection rate fluctuated at a level of 22%. The lowest rate was 21.75% in the third year after the surgery, and the highest rate was 23.5% in the fifth year after the surgery (**Figure 2A**). The majority were found to be single HPV infection (858/1203, 71.32%) and fluctuated around 17.58% annually, as shown in **Figure 2B**. The main HPV genotypes were HPV 16 (177/1203, 14.71%), HPV 39 (104/1203, 8.65%), HPV 52 (187/1203, 15.44%), HPV 53 (171/1203, 14.21%), HPV 58 (105/1203, 8.73%), and HPV 81 (126/1203, 10.47%) in the cohort (**Figure 3**). We also found that α-9 HPV infection was significantly associated with multiple HPV genotype infection (OR: 5.40, 95%CI:4.10-7.12, p<.001).

10.2% (411/4017) of the patients were found with sequela vaginal lesions, with 320 (77.86%) cases of LSIL, 76 (18.49%) cases of HSIL, and 15 (3.65%) with vaginal carcinoma. Particular to each year, the prevalence rate of vaginal lesions gradually decreased from 6.97% at Y1 to 2.96% at Y5, mainly due to the declining prevalence of LSIL from 5.27% to 2.22% (**Figure 2C**). Vaginal HSIL decreased sharply in the first three years, after which the prevalence remained at a low level of 0.5%. As for the prevalence rate of vaginal cancer, it remained low, fluctuating at 0.1%.

### 3.3 Clinicopathological risk factors for sequela vaginal lesions

**Figure 4:**
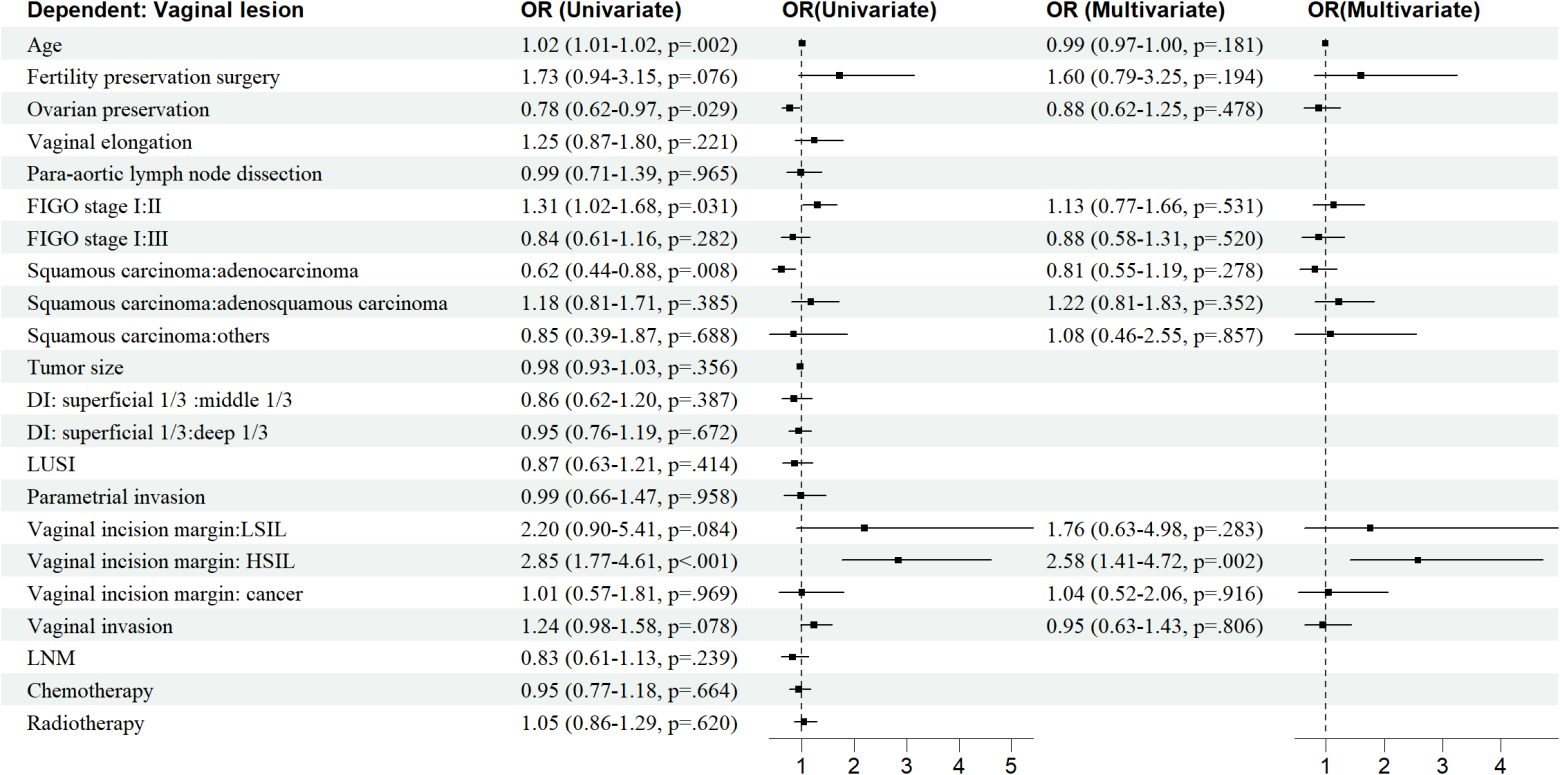
Nomogram of predicting vaginal lesions with factors from multivariate analysis.

Age, OP, FIGO stage, histological type, vaginal incision margin, and HPV infection were found to significantly influence the incidence of sequela vaginal lesion (P<0.05), as shown in **Table 1**. As can be seen in **Figure 4**, patients who were older (OR = 1.02, 95% CI: 1.01–1.02) or at stage II (OR = 1.31, 95% CI: 1.02–1.68) were more susceptible to developing vaginal lesions. OP during surgery (OR = 0.78, 95% CI: 0.62–0.97) and histology as adenocarcinoma (OR = 0.62, 95% CI: 0.44–0.88) seemed to be protective from further vaginal lesions. As for vaginal incision margin, compared with a negative margin, both LSIL status (OR = 2.20, 95% CI: 0.90–5.41) and HSIL status (OR = 2.85, 95% CI: 1.77–4.61) were remarkably related to vaginal lesions. However, an incision margin with cancer (OR = 1.01, 95% CI: 0.57–1.81) was insignificantly related to vaginal lesions.

**Figure 5:**
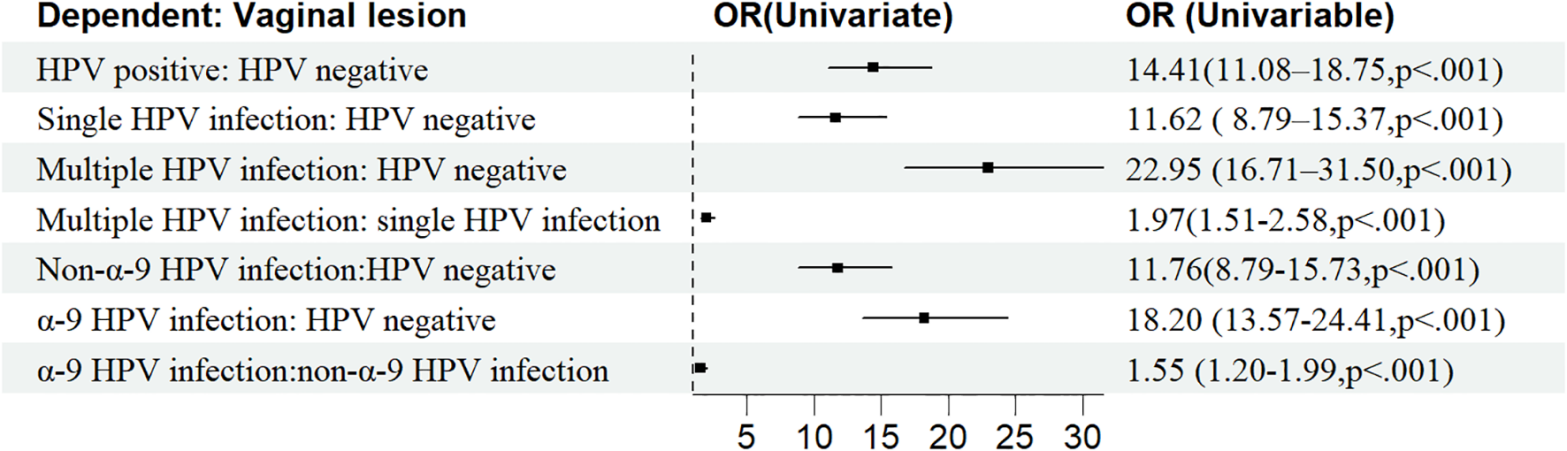
Clinicopathological risk factors associated with vaginal disease progression after surgical treatment.

**Figure 6:**
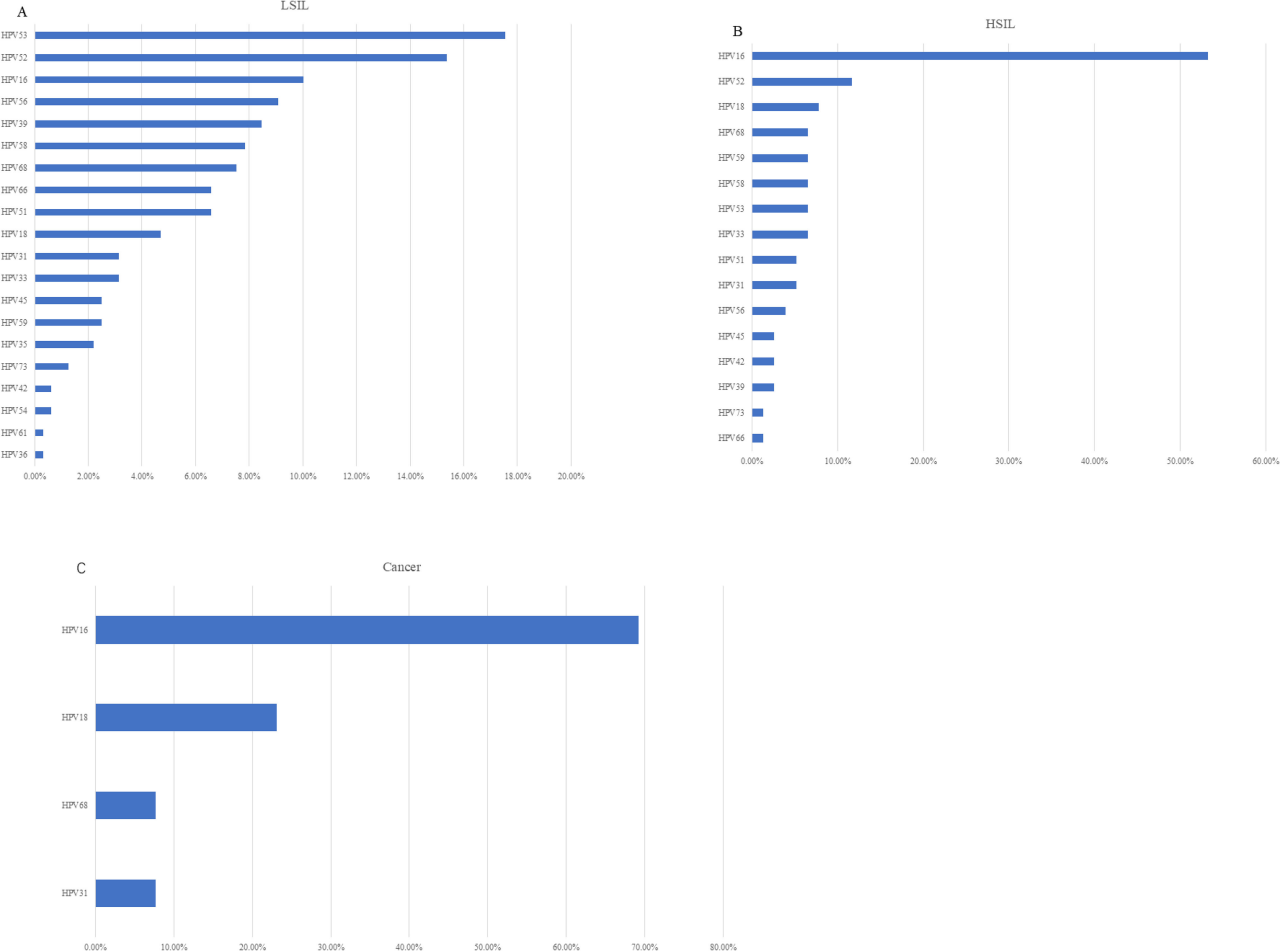
**(A)** HPV infection rate and the incidence of vaginal lesions within five years post-operation **(B)** Single HPV infection rate and multiple HPV infection rate within five years post-operation **(C)** The incidence of LSIL, HSIL, and vaginal cancer within five years post-operation.

HPV infection turned out to be the most critical risk factor for vaginal lesions (OR = 14.41, 95% CI: 11.08–18.75) (**Figure 5**). Both single HPV infection (OR = 11.62, 95% CI: 8.79–15.37) and multiple HPV infection (OR = 22.95, 95% CI: 16.71–31.50) strongly induced the development of vaginal lesions, and multiple HPV genotype had more impact than single HPV genotype regardless of HPV titration (OR = 1.97, 95% CI: 1.51-2.58). Then we focused on the HPV genotype and found both non-α-9 HPV infection (OR = 11.76, 95% CI: 8.79-15.73) and α-9 HPV infection (OR = 18.20, 95% CI: 13.57-24.41) could cause the development of vaginal lesions, with α-9 HPV infection involving higher risk than non α-9 types (OR = 1.55, 95% CI: 1.20-1.99). The prevalence of a specific HPV genotype might significantly affect the progression of vaginal lesions, and HPV 16 and 18 were severely associated with HSIL and cancer while HPV 53 and 52 were more detected in vaginal LSIL (**Figure 3**). Specific vaginal lesions with each genotype of HPV are displayed in **Figure 6**; the α-9 HPV genotype was predominant in patients with HISL (83.12%).

For the HPV kit, sensitivity of the test was 104 copies/mL (20 copies/reaction), for which low titration of HPV infection is presented as negative. To continuously observe the status of patients with HPV turning negative, patients with HPV normalized in the first year were followed up for the next five years after major surgery (**Table 2**). Patients with HPV that turned negative after the first year had a 50% risk of turning back positive each year, till only 73 patients were found to be negative for five years in a row. Of these patients, two were found with LSIL at Y3. Analysis of this population revealed that there is no significant difference in their risk of getting vaginal lesions as to the length of negative HPV duration (P>0.01).

**Table 2:** Continuous follow-up of HPV negative patients at Y1.

HPV negative duration	One year	Two years	Three years	Four years	Five years
Number	1468	655	334	155	73
With vaginal lesion	73	24	20	4	2
Without vaginal lesion	1395	631	314	151	71
P	/	0.222	0.5353	0.2569	0.5574

Further multivariate logistic analysis showed HSIL incision margin (OR = 2.58, 95% CI: 1.41–4.72), single non-α-9 HPV infection (OR = 11. 08, 95% CI: 8.17–15.02), single α-9 HPV infection (OR = 12. 58, 95% CI: 8.79–18.00), multiple HPV infection without α-9 HPV (OR = 16.30, 95% CI: 9.91–26.81), and multiple HPV infection with α-9 HPV (OR = 26.50, 95% CI: 18.66–37.65) remained remarkably associated with vaginal lesions (P<0.05). As multiple HPV infection was significantly associated with α-9 HPV infection, patients with α-9 HPV infection were more prone to get multiple HPV infections (OR = 1.55, 95% CI: 1.20–1.99); these factors were unified as HPV-infection.

### 3.4 Construction of the predictive model for sequela vaginal lesion in patients with cervical cancer treated with major surgery

“Caret” R package was used to randomly divide 4017 patients into a training cohort (2813 patients) to establish the model and a testing cohort (1204 patients) to verify the model. There was no significant difference in clinicopathological characteristics between the two cohorts (**Supplementary Table 1**). Due to the co-linearity between multiple HPV infections and α-9 HPV genotype infection, they were taken as one variable: HPV infection. Then machine learning was performed with five methods, and univariable and multivariable logistic analysis, LASSO regression, stepwise regression, optimum subset regression, and random forest regression were performed to screen for the risk factors to build a model with training data.

Fivefold cross validation was used in LASSO regression and HPV infection, from which vaginal elongation and the vaginal incision margin were finally picked to predict the probability of vaginal lesions. Fivefold cross validation was also applied in Random Forest regression to adjust optimal parameters (mtry=4, ntree=200). Then we ordered the risk factors according to their importance, and age, tumor size, and HPV infection were the top three risk factors in this prediction model calculation. Vaginal elongation, incision margin status, and HPV infection were selected in a stepwise regression model (P<0.1) and in an optimum subset regression model according to Mallows’Cp. For the multivariate logistic regression model, the vaginal incision margin and HPV infection were picked as critical factors for prediction. Therefore, three predictive models were further constructed: Model 1 including HPV infection, vaginal elongation, and vaginal incision margin; Model 2 involving age, tumor size, and HPV infection; and Model 3 with vaginal incision margin and HPV infection.

### 3.5 Accuracy and security verification of the three predictive models

**Figure 7:**
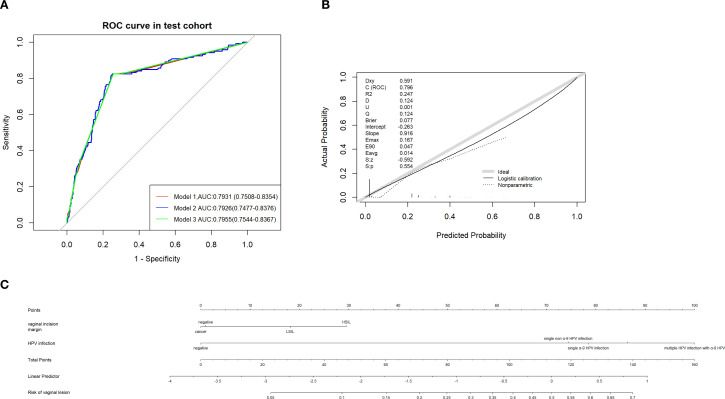
**(A)** ROC of different predictive models established in predicting vaginal lesions and AUC were compared. **(B)** Calibration curves of predictive models above. **(C)** Nomogram of predicting vaginal lesion with factors from multivariate analysis respectively.

Considering the potential overdiagnosis and underdiagnosis of vaginal lesions in this patient population, the low-risk group predicted by the model who are recommended to observe with no further interference must be definite. ROC of the three models was used and Model 3 had the maximal AUC (0.7955, 95% CI:0.7544-0.8367) compared with model 1 (0.7931, 95% CI:0.7508-0.8354) and model 2 (0.79226, 95% CI:0.7477-0.8376) (**Figure 7A**). Judging from the degree of agreement with the 45°curve and the value of Brier, D, U, and Q in the calibration curve, model 3 was still the optimal model (**Supplementary Table 2**, **Figure 7B**). DCA (**Supplementary Figure 1**) was drawn to further prove the effectiveness of Model 3, and the logistic regression model was still the optional model. With Model 3 (multivariate logistic regression model) predicting the risk of vaginal lesions (**Figure 7C**), we found that risk for patients without sequela vaginal lesions in our cohort was 2.52% and risk for those with vaginal lesions was 25.81%, which proved the effectiveness and security of this predictive model.

## 4 Discussion

Our results indicated that cervical cancer patients after hysterectomy with persistent HPV infection are at an increased risk of vaginal lesions, especially those with multiple α-9 HPV infection. Women with cervical SCC are more prone to VaAIN/recurrence, especially within the first three years after hysterectomy. Intraoperative ovarian preservation and adenocarcinoma pathology were found to be protective from sequela vaginal lesion, while elder age, stage II, and a positive incisional margin were high-risk factors for vaginal lesions. A predictive model was developed in our study to help identify high-risk populations of vaginal lesions and guide our clinical interventions.

During the five years of follow-up after undergoing major surgery for cervical cancer, 70% of the patients successfully eradicated HPV infection, while the remaining patients exhibited persistent or intermittent HPV infection at a random year, with nearly all of them being infected with high-risk HPV types. Compared with research about HPV infection genotype before surgery (8), we found that the major infection genotype after surgery was α-9 HPV infection (HPV 16, 31, 33, 35, 52, 58, 67) and HPV18 infection was highly likely to be cured after surgery. The difference might be because of the removal of the cervix region. The same result was also found in other research (9).

29.94% of patients continued to maintain HPV infection within five years after surgery, with a persistent positive rate of 22% each year. This result is close but slightly higher than previous studies, showing the persistent HPV infection rate after hysterectomy for patients with a history of CIN as 12.4% (10/81) (10), while another domestic study reported the rate as 18.8% (157/834) (11). This may be because our study included a higher percentage of cervical cancer patients, and that patients with cervical carcinoma usually possess an HPV infection with a higher viral load. Our previous data indicated that the higher the HPV viral load, the harder it was for patients to finally eradicate the infection (12).

Our study revealed the trend of HPV clearance rate post-operation remained stable at around 22-23% (**Figure 2A**) within five years after surgery; previous studies reported that HPV was usually rapidly eliminated at 6 to 9 months after surgery with a median clearance duration at 10.4 months (12, 13). We shall conclude that, if a patient failed to eradicate HPV infection within one year post-operation, it is very likely that they would be persistently infected as a high-risk population for sequela lesions.

This study firstly conducted a continuous five-year follow-up on patients’ HPV status and vaginal lesions and revealed an incidence of sequela vaginal lesion at 10.2% (411/4018), which is lower than previously reported. In another study, 13.6% (20/147) of patients developed vaginal lesions after hysterectomy (6). The difference may be that our patients possibly benefited from the superior follow-up system in our center. Vaginal lesions were found to mainly occur within the first three years post-operation, then there is a remarkable annual decline in the incidence of vaginal lesions (**Figure 2A**), mainly due to the decrease of LSIL and HSIL. Incidence of HSIL and vaginal carcinoma remained low, which could be a result of early detection and prompt interference.

Our data demonstrated that ovarian preservation during hysterectomy was a protective factor against subsequent vaginal lesions (OR = 0.78, 95% CI: 0.62–0.97), possibly mediated through the production of estrogen. A previous study has shown high rates of regression and cure among VaIN patients treated with intravaginal estrogen, whether alone (90.0%) or in combination with other modalities (81.3%) (14). Estrogen was proved to activate the immune response by regulating immune cells, such as neutrophils, natural killer cells, macrophages, and monocytes, through nuclear estrogen receptor (nERs), including ERα and ERβ, and the membrane-bound estrogen receptor, G-protein-coupled estrogen receptor (GPER) (15). Our investigation also suggested the massive benefits of secure ovarian preservation for selected patients during surgery, not only to preserve endogenous hormone production but also to support long-term gynecologic health. This conclusion also gave us a clue as to why older patients presented with worse viral elimination and higher risk for vaginal lesions and disease recurrence.

In our study, histology as adenocarcinoma was found to be a protective factor (OR = 0.62, 95% CI: 0.44–0.88), and the cervical SCC is a risk factor for consequent vaginal epithelial lesions according to a previous study (6). As we know, most cervical epithelial carcinomas (SCC, adenocarcinoma, and adenoSCC) were due to pathogenic HPV infection; our data might provide an indirect insight into a diverse pathogenic mechanism between SCC and adenocarcinoma in which different HPV genotypes may tend to infect different histological cells, thereby triggering different mechanisms that lead to the occurrence of cervical cancer. α-9 HPV genotypes are more likely to infect squamous cells while α-7 HPV genotypes are more likely to infect adenoid cells. So, most patients infected with α-9 HPV genotypes retained HPV infection of the vaginal squamous epithelium after surgery. Vaginal intraepithelial lesions might follow the same mechanism as cervical SCC. Also, the vaginal epithelium consists of squamous cells, and it is difficult for ADC to invade vaginal tissue. More investigations are warranted to confirm this observation.

A positive vaginal incision margin with LSIL or HSIL was found to be a significant risk factor. According to our data, a positive surgical margin with cancer was considered insignificant on consequent vaginal lesions. All these patients accepted vaginal brachytherapy (VBT) as adjuvant therapy, while for patients with LSIL and HSIL, carbon dioxide laser treatment was given. Adjuvant VBT has been proven to largely reduce the risk of further vaginal lesions s (16).

A retrospective study revealed that multifocal lesions was a significant predictor for VAIN recurrences, perhaps due to residual lesions (17). Previous studies reported 84–96% of cases of VaIN II/III/vHSIL were positive for HPV, and HPV infection rates increased with the severity of VaIN (18, 19). Multiple studies have proved HPV, as an etiologic agent, could infect the vaginal epithelium and promote vaginal lesions, similar to the pathogenesis of cervical intraepithelial neoplasia (CIN) (9, 20), and our study further revealed multiple HPV infections and α-9 HPV infection could accelerate the pathogenesis of vaginal lesions.

Almost 1/3 of the patients had negative HPV at the first year post-operation, while continuous observation of this population revealed that they held a 50% risk of HPV returning each year. Patients with negative HPV five years in a row still had a chance to develop vaginal lesions. In this case, women with risk factors for VaIN, especially those with a hysterectomy for HPV-related disease, held an increased risk for vaginal lesions, so routine application of cytology and HR HPV co-testing is required for continuous surveillance and detection of potential vaginal lesions, even when HPV has been negative for a long time. If the results of a screen cytology and HPV co-test are suspicious, our predictive model could predict the risk of vaginal lesions to a certain extent using only two parameters: the incision margin status of the previous major surgery and the HPV infection status. With these, we could potentially prevent unnecessary colposcopic examinations and relieve patients’ anxiety under this clinical scenario. With our model utilized in this study population, the vaginal disease risk was calculated as 2.52% in patients with no lesions detected and 25.81% with positive lesions found. According to this, when the risk calculated is greater than 25%, it is inferred that patients are extremely high risk, and proactive measures should be initiated immediately. When the risk calculated is less than 2%, the disease risk is considered low and further follow-up can be conducted. When it falls within the range of 2% to 25% for risk of vaginal lesions, whether to undertake further colposcopic exams can be individualized and decided with the gynecology oncologist and the patient together.

The results of our analysis suggest that surgical margin status could confer sufficient elevated risk of recurrence to warrant frequent screening and further colposcope examinations in post-hysterectomy patients, and the risk differs by HPV infection greatly. Our cohort presented a very high threshold of >25% recurrence risk, while our tool offered a potential in consideration of lower thresholds than this. This more contemporary approach may provide a more tailored and effective tool for clinicians to base colposcopic recommendations to their patients individually. In our clinical setting, risk threshold between 2-25% was acceptable and these risks should be carefully communicated with patients before decisions made using this predictive model.

Although our predictive model is optimized through machine learning, it still has some limitations. Our study was based on a single-center, retrospective study, and a further multi-center prospective randomized control trial is needed to help validate the model. To maintain cohort integrity, prevent exclusion bias, and reflect real-world management pathways where diagnostic escalation is not always pursued, the result of LCT was taken as the condition of vaginal epithelium if patients refused to undergo colposcopy. Patient-reported quality-of-life metrics were not assessed; future studies should evaluate this potential benefit.

This study represents the first large-scale analysis to investigate the risk factors for subsequent vaginal lesions specifically in patients with cervical cancer who have undergone hysterectomy or fertility-sparing treatment. Furthermore, it is the largest retrospective study to evaluate vaginal lesions in the post-hysterectomy population. Our study constructed a bivariate predictive model through machine learning with a decent sample size of 4017 patients. The model could help guide gynecology oncologists to rapidly make clinical decisions on whether to follow-up or further examine patients with more interventions.

## Data Availability

The original contributions presented in the study are included in the article/**Supplementary Material**. Further inquiries can be directed to the corresponding author.

## References

[1] Ferlay J Soerjomataram I Dikshit R Eser S Mathers C Rebelo M. Cancer incidence and mortality worldwide: sources, methods and major patterns in GLOBOCAN 2012. Int J Cancer. (2015) 136:E359–86. doi: 10.1002/ijc.29210, PMID: 25220842

[2] Zheng RS Chen R Han BF Wang S Sun K Chen R. Cancer incidence and mortality in China, 2022. Zhonghua Zhong Liu Za Zhi. (2024) 46:221–31. doi: 10.3760/cma.j.cn112152-20240119-00035, PMID: 38468501

[3] Li J Ouyang X Gong X Li P Xiao L Chang X. Survival outcomes of minimally invasive surgery for early-staged cervical cancer: A retrospective study from a single surgeon in a single center. Asian J Surg. (2022) 45:320–5. doi: 10.1016/j.asjsur.2021.05.037, PMID: 34148755

[4] Elit L Fyles AW Oliver TK Devries-Aboud MC Fung-Kee-Fung M members of the Gynecology Cancer Disease Site Group of Cancer Care Ontario’s Program in Evidence-Based Care. Follow-up for women after treatment for cervical cancer. Curr Oncol. (2010) 17:65–9. doi: 10.3747/co.v17i3.514, PMID: 20567627 PMC2880906

[5] Iacobone AD Radice D Guerrieri ME Spolti N Grossi B Bottari F. Which risk factors and colposcopic patterns are predictive for high-grade VAIN? A retrospective analysis. Diagnostics (Basel). (2023) 13(2):176. doi: 10.3390/diagnostics13020176, PMID: 36672986 PMC9858341

[6] Li Z Barron S Hong W Karunamurthy A Zhao C. Surveillance for recurrent cancers and vaginal epithelial lesions in patients with invasive cervical cancer after hysterectomy: are vaginal cytology and high-risk human papillomavirus testing useful? Am J Clin Pathol. (2013) 140:708–14. doi: 10.1309/ajcph4afszhu8ekk, PMID: 24124151

[7] Kesic V Carcopino X Preti M Vieira-Baptista P Bevilacqua F Bornstein J. The European Society of Gynaecological Oncology (ESGO), the International Society for the Study of Vulvovaginal Disease (ISSVD), the European College for the Study of Vulval Disease (ECSVD), and the European Federation for Colposcopy (EFC) consensus statement on the management of vaginal intraepithelial neoplasia. Int J Gynecol Cancer. (2023) 33:446–61. doi: 10.1136/ijgc-2022-004213, PMID: 36958755 PMC10086489

[8] Yang X Li Y Tang Y Li Z Wang S Luo X. Cervical HPV infection in Guangzhou, China: an epidemiological study of 198,111 women from 2015 to 2021. Emerg Microbes Infect. (2023) 12:e2176009. doi: 10.1080/22221751.2023.2176009, PMID: 36744409 PMC9936994

[9] Zhou FY Zhou Q Zhu ZY Hua KQ Chen LM Ding JX. Types and viral load of human papillomavirus, and vaginal microbiota in vaginal intraepithelial neoplasia: a cross-sectional study. Ann Transl Med. (2020) 8:1408. doi: 10.21037/atm-20-622, PMID: 33313153 PMC7723660

[10] Bruno MT Panella MM Valenti G Di Grazia S Sgalambro F Farina J. Vaginal intraepithelial neoplasia (VaIN) after hysterectomy is strongly associated with persistent HR-HPV infection. Cancers (Basel). (2024) 16(14):2524. doi: 10.3390/cancers16142524, PMID: 39061164 PMC11274675

[11] Cao D Wu D Xu Y. Vaginal intraepithelial neoplasia in patients after total hysterectomy. Curr Probl Cancer. (2021) 45:100687. doi: 10.1016/j.currproblcancer.2020.100687, PMID: 33309077

[12] Wang Q Zhou FY Ding JX. Factors associated with the persistence of human papillomavirus after surgery in patients with cervical cancer. Diagn Microbiol Infect Dis. (2024) 108:116201. doi: 10.1016/j.diagmicrobio.2024.116201, PMID: 38340484

[13] Abudurexiti G Tuerxun G Abulizi G Mijiti P Aierken K Maimaiti A. Human papillomavirus posttreatment clearance time in cervical intraepithelial neoplasia and invasive cervical cancer. J Low Genit Tract Dis. (2020) 24:34–7. doi: 10.1097/lgt.0000000000000495, PMID: 31725049 PMC6924945

[14] Rhodes HE Chenevert L Munsell M. Vaginal intraepithelial neoplasia (VaIN 2/3): comparing clinical outcomes of treatment with intravaginal estrogen. J Low Genit Tract Dis. (2014) 18:115–21. doi: 10.1097/LGT.0b013e31829f52f4, PMID: 24189311

[15] Hoffmann JP Liu JA Seddu K Klein SL. Sex hormone signaling and regulation of immune function. Immunity. (2023) 56:2472–91. doi: 10.1016/j.immuni.2023.10.008, PMID: 37967530

[16] Sedlis A Bundy BN Rotman MZ Lentz SS Muderspach LI Zaino RJ. A randomized trial of pelvic radiation therapy versus no further therapy in selected patients with stage IB carcinoma of the cervix after radical hysterectomy and pelvic lymphadenectomy: A Gynecologic Oncology Group Study. Gynecol Oncol. (1999) 73:177–83. doi: 10.1006/gyno.1999.5387, PMID: 10329031

[17] Dodge JA Eltabbakh GH Mount SL Walker RP Morgan A. Clinical features and risk of recurrence among patients with vaginal intraepithelial neoplasia. Gynecol Oncol. (2001) 83:363–9. doi: 10.1006/gyno.2001.6401, PMID: 11606098

[18] Stuebs FA Koch MC Mehlhorn G Gass P Schulmeyer CE Hartman A. Accuracy of colposcopic findings in detecting vaginal intraepithelial neoplasia: a retrospective study. Arch Gynecol Obstet. (2020) 301:769–77. doi: 10.1007/s00404-020-05441-5, PMID: 31993733

[19] De Vuyst H Clifford GM Nascimento MC Madeleine MM Franceschi S. Prevalence and type distribution of human papillomavirus in carcinoma and intraepithelial neoplasia of the vulva, vagina and anus: a meta-analysis. Int J Cancer. (2009) 124:1626–36. doi: 10.1002/ijc.24116, PMID: 19115209

[20] Sugase M Matsukura T. Distinct manifestations of human papillomaviruses in the vagina. Int J Cancer. (1997) 72:412–5. doi: 10.1002/(SICI)1097-0215(19970729)72:3<412::AID-IJC7>3.0.CO;2-S, PMID: 9247283

