# Supplementary material for: A novel bivariate nomogram for predicting sequela vaginal lesions after surgery in patients with HPV-associated cervical cancer
Source: Front Oncol. 2025 Sep 26;15:1587520. doi: 10.3389/fonc.2025.1587520 (PMC12510852; doi:10.3389/fonc.2025.1587520)
Supplement: Supplementary file 1 [file DataSheet1.docx]

Supplementary Material

# Supplement Table 1: The comparison of test cohort and train cohor

|  | Test cohort | Train cohort | P |
| --- | --- | --- | --- |
| N | 1204 | 2813 |  |
| Age (year)(median) | 48.7 (10.6) | 48.4 (10.5) | 0.407 |
| Fertility-sparing surgery(%) |  |  | 0.898 |
| No | 1179 (97.9%) | 2758 (98.0%) | |
| Yes | 25 (2.08%) | 55 (1.96%) |  |
| Ovarian preservation (%) |  |  | 0.798 |
| No | 798 (66.3%) | 1851 (65.8%) |  |
| Yes | 406 (33.7%) | 962 (34.2%) |  |
| Vaginal elongation |  |  | 0.313 |
| No | 1122 (93.2%) | 2594 (92.2%) |  |
| Yes | 82 (6.81%) | 219 (7.79%) |  |
| FIGO stage |  |  | 0.489 |
| Ⅰ | 806 (66.9%) | 1875 (66.7%) |  |
| Ⅱ | 219 (18.2%) | 549 (19.5%) |  |
| Ⅲ | 179 (14.9%) | 389 (13.8%) |  |
| Para-aortic lymph node dissection (%) |  |  | 0.974 |
| No | 1081 (89.8%) | 2523 (89.7%) |  |
| Yes | 123 (10.2%) | 290 (10.3%) |  |
| Histology (%) |  |  | 0.257 |
| Squamous carcinoma | 942 (78.2%) | 2166 (77.0%) |  |
| Adenocarcinoma | 164 (13.6%) | 385 (13.7%) |  |
| Adenosquamous carcinoma | 83 (6.89%) | 201 (7.15%) |  |
| Others | 15 (1.25%) | 61 (2.17%) |  |
| DI |  |  | 0.352 |
| Superficial 1/3 | 620 (51.5%) | 1455 (51.7%) |  |
| Middle 1/3 | 140 (11.6%) | 368 (13.1%) |  |
| Deep 1/3 | 444 (36.9%) | 990 (35.2%) |  |
| Tumor size (cm)(median) | 1.87 (2.01) | 1.89 (1.97) | 0.774 |
| LUSI |  |  | 0.626 |
| Negative | 1052 (87.4%) | 2475 (88.0%) |  |
| Positive | 152 (12.6%) | 338 (12.0%) |  |
| Parametrial invasion (%) |  |  | 0.613 |
| Negative | 1114 (92.5%) | 2617 (93.0%) |  |
| Positive | 90 (7.48%) | 196 (6.97%) |  |
| Vaginal incision margin status (%) |  |  | 0.593 |
| Negative | 1131 (93.9%) | 2627 (93.4%) |  |
| LSIL | 11 (0.91%) | 20 (0.71%) |  |
| HSIL | 29 (2.41%) | 68 (2.42%) |  |
| Cancer | 33 (2.74%) | 98 (3.48%) |  |
| Vaginal invasion (%) |  |  | 0.505 |
| Negative | 968 (80.4%) | 2234 (79.4%) |  |
| Positive | 236 (19.6%) | 579 (20.6%) |  |
| LNM (%) |  |  | 0.44 |
| Negative | 1023 (85.0%) | 2418 (86.0%) |  |
| Positive | 181 (15.0%) | 395 (14.0%) |  |
| HPV infection(%) |  |  | 0.11 |
| Negative | 829 (68.9%) | 1985 (70.6%) |  |
| Non α-9 single HPV infection | 167 (13.9%) | 410 (14.6%) |  |
| α-9 single HPV infection | 93 (7.72%) | 188 (6.68%) |  |
| Multiple HPV infection without α-9 HPV | 39 (3.24%) | 56 (1.99%) |  |
| Multiple HPV infection with α-9 HPV | 76 (6.31%) | 174 (6.19%) |  |
| TCT |  |  | 0.675 |
| Without vaginal lesion | 1085 (90.1%) | 2521 (89.6%) |  |
| With vaginal lesion | 119 (9.88%) | 292 (10.4%) |  |
| Chemotherapy (%) |  |  | 0.808 |
| Unreceived | 740 (61.5%) | 1742 (61.9%) |  |
| Received | 464 (38.5%) | 1071 (38.1%) |  |
| Radiotherapy (%) |  |  | 0.777 |
| Unreceived | 683 (56.7%) | 1611 (57.3%) |  |
| Received | 521 (43.3%) | 1202 (42.7%) |  |

# Supplement Table 2: The value of Brier, D, U and Q in every model

|  | Brier | D | U | Q |
| --- | --- | --- | --- | --- |
| Model 1 | 0.077 | 0.122 | 0.001 | 0.122 |
| Model 2 | 0.077 | 0.126 | 0.000 | 0.126 |
| Model 3 | 0.077 | 0.124 | 0.001 | 0.124 |

## Supplementary Figures

**
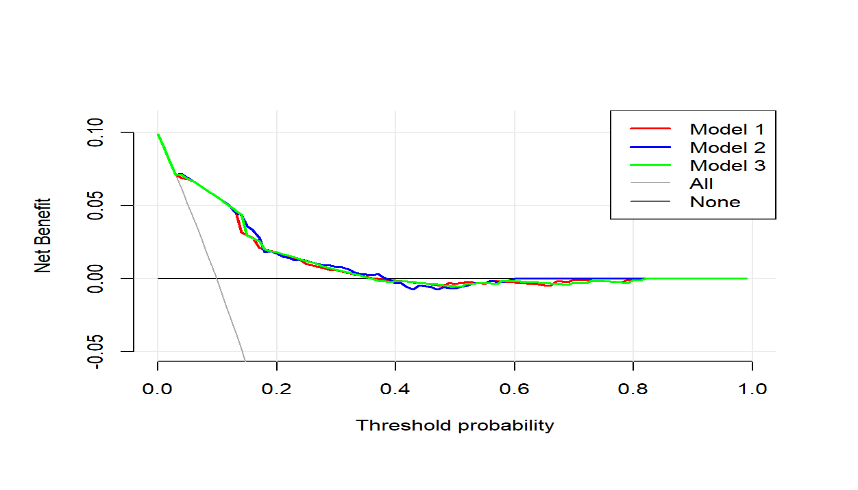
**

**Supplementary Figure 1.** The decision curve analysis (DCA) of three models in test cohort
